# Accelerated corneal cross-linking (18mW/cm2 for 5 min) with HPMC-riboflavin in progressive keratoconus – 5 years follow-up

**DOI:** 10.1007/s00417-023-06225-8

**Published:** 2023-09-06

**Authors:** Julia Friedrich, Alexandra Sandner, Ali Nasseri, Mathias Maier, Daniel Zapp

**Affiliations:** 1 Klinik und Poliklinik für Augenheilkunde, Klinikum rechts der Isar, TUM, Munich, Germany; 2 Technische Universität München, Munich, Germany

**Keywords:** Corneal crosslinking, Keratoconus, Keratectasia, Accelerated crosslinking, 18mW, Cornea

## Abstract

**Purpose:**

To evaluate long-term results of accelerated corneal cross-linking (ACXL) in patients with progressive keratoconus, seventy-four eyes of 53 patients with progressive keratoconus (documented Kmax progression > 1D/a) who underwent ACXL (18mW/cm2 for 5 min) were included in a retrospective observational clinical study. The investigation focused on tomographic and keratometric parameters, refractive data, and visual outcomes at 5 years follow-ups.

**Methods:**

Corrected distance visual acuity (CDVA), slit lamp, and Pentacam® examinations were conducted, including assessments of thinnest corneal point (TP), minimum radius (Rmin), corneal astigmatism, and maximum anterior keratometry (Kmax). These examinations were performed two weeks before the surgery and, on average, 56 months after the surgery. In a subgroup of 24 eyes, Pentacam® examination data from an intermediate visit at 12 months until the final visit was evaluated to confirm continuous stability.

The ACXL protocol included corneal abrasion, hydroxypropylmethylcellulose (HPMC)-riboflavin eye drops administered every 5 min for a total duration of 30 min, and irradiation with 18mW/cm2 for 5 min using riboflavin eye drops applied every minute during the irradiation process. Intraoperatively, minimal corneal pachymetry of > 400 µm was ensured in every patient.

**Results:**

After 56 months, all values exhibited statistically significant changes (paired t-test; CDVA *p* = 0.002; Kmax *p* < 0.001; Rmin *p* < 0.001; astigmatism *p* = 0.03; TP *p* < 0.001).

In the subgroup analysis of 24 eyes, which included tomographical and keratometric parameters, no statistically significant changes were observed during the last 12 months of observation (paired t-test; Kmax *p* = 0.72; Rmin *p* = 0.67; astigmatism *p* = 0.72).

Treatment failure was strictly defined as an increase in Kmax (> 1D) during the 5-year follow-up and was observed in only 3 eyes (4%).

**Conclusions:**

ACXL is an effective and safe treatment for patients with progressive keratoconus. Our results demonstrate improvements in functional and tomographical outcomes even after high-energy ACXL (18mW/cm2 for 5 min) over a long-term period of 56 months. Our analysis indicates stable conditions in previously progressive keratoconus, particularly during the final year of the observation period. The treatment failure rate was 4%. 
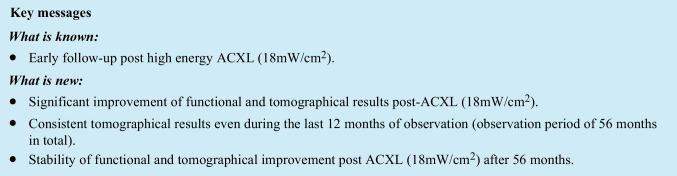

## Introduction

The group of ectatic diseases includes keratoconus, pellucid marginal degeneration, keratoglobus, and post-refractive surgery progressive corneal ectasia. The pathophysiology of keratoconus is multifactorial and involves genetic, environmental, biomechanical, and biochemical factors [1]. The mean age at diagnosis is reported to be 28 years, with an incidence of 1:7500 [2].

Disease-defining parameters of ectatic diseases include corneal thinning, posterior or combined posterior-anterior ectasia, and abnormal corneal thickness distribution [1, 3, 4]. These parameters are best diagnosed using tomographical examinations such as Scheimpflug or optical coherence tomography [4].

More recent corneal tomography techniques allow for the confirmation of corneal ectasia at a very early stage, enabling the diagnosis of even mild or subclinical keratoconus [4, 5]. This advancement enables ophthalmologists to initiate therapy before severe irregular astigmatism of the cornea, corneal scarring, or Descemet's membrane rupture can lead to impaired vision [3].

Classic and symptomatic treatment include refractive correction using gas-permeable contact lenses, special cone-design lenses, soft lenses, and even scleral and hybrid lenses [6].

The primary goal of interventional keratoconus therapy is to halt disease progression, making early treatment crucial for better long-term visual acuity [5]. However, in patients with good baseline visual acuity, a thorough risk–benefit analysis of interventional treatment is essential. Modified techniques of Corneal Crosslinking (CXL), known as accelerated Corneal Crosslinking (ACXL), have been developed to reduce the instantaneous complications associated with conventional CXL techniques, such as endothelial-related side effects and time-related discomfort. Consequently, the timing of intervention has gradually shifted to earlier disease stages [4, 7]. Nonetheless, there is an ongoing discussion regarding the efficacy of ACXL compared to traditional CXL.

The purpose of this monocentric exploratory study is to evaluate the long-term results of ACXL (18mW/cm2 for 5 min) in patients with progressive keratoconus.

## Material and methods

The original Dresden protocol for corneal cross-linking (CXL) involves central corneal abrasion and the application of photosensitizing 0.1% riboflavin (10 mg riboflavin-5-phosphate in 10 ml Dextran-T-500 20% solution) eye drops. Once the riboflavin has completely penetrated the cornea, irradiation is initiated and continued for 30 min using UVA light at a power density of 3mW/cm2. During irradiation, riboflavin eye drops are applied every 5 min, resulting in a total dose of 5.4 J/cm2 [8].

The ACXL protocol investigated in this study involved sterile mechanical abrasion of the central corneal epithelium (epi-off CXL) after local anesthesia. Subsequently, riboflavin eye drops (0.1% riboflavin in hydroxypropyl-methylcellulose = HPMC; VibeX Rapid; Avedro Inc.) were applied every 4–5 min for 30 min. Prior to irradiation, minimal corneal pachymetry of > 400 µm was ensured in every patient by using a hypoosmolar solution (MedioCROSS® H, Avedro Inc.) to swell the cornea for 2 min every 20 s in case the corneal thickness was < 400 µm at the thinnest corneal point (TP) until a sufficient pachymetry > 400 µm was achieved. Corneal irradiation was performed using a power density of 18mW/cm2 (CCL 365 vario, Peschke, Meditrade, Huenenberg, Switzerland) for 5 min, resulting in a total dose of 5.4 J/cm2. During irradiation, iso-osmolar riboflavin eye drops were applied every minute, and the limbal region was excluded. Finally, a sterile contact lens was applied in combination with levofloxacin eye drops.

A total of 74 eyes from 53 patients with documented progressive keratoconus were included in a retrospective observational clinical study. Progressive keratoconus was defined as the annual progression of the steepest radius of curvature of the anterior corneal surface (maximum anterior keratometry = Kmax) exceeding 1 diopter (D) [9]. The patients underwent accelerated corneal cross-linking (ACXL) at the Department of Ophthalmology, Klinikum rechts der Isar, Technical University of Munich (TUM), between February 2013 and June 2017. The ACXL procedure involved UVA radiation (365 nm, CCL-365 vario, Peschke Meditrade, Huenenberg, Switzerland) at a power density of 18mW/cm2 for a duration of 5 min.

Exclusion criteria were defined as TP < 400 µm, post-LASIK ectasia, pellucid marginal degeneration, corneal infection, corneal scars that could interfere with tomographical examination, prior corneal surgery, prior penetrating trauma, prior rupture of the Descemet’s membrane, additional diseases such as glaucoma or aphakia, pregnancy, and breastfeeding [10].

Written consent according to the principles of the Declaration of Helsinki was received by all patients.

The investigation focused on tomographical and keratometric parameters, refractive data, and visual outcomes during the 5-year follow-up period. Demographic values including gender, age, localization, and bilateralism were collected pre-interventionally. Corrected distance visual acuity (CDVA), slit lamp exams, and tomographical examinations were performed two weeks before and on average 56 months after surgery. Tomographical examination, using a Scheimpflug device (Pentacam® HR 70700, Oculus, Wetzler, Germany) included the thinnest corneal point (TP), minimum anterior radius (Rmin), corneal astigmatism and maximum anterior keratometry (Kmax). Pentacam® examination data from an intermediate visit conducted 12 months before the patient's final visit were evaluated in a subgroup of 24 eyes. This analysis aimed to confirm the continuous stability of the parameters, both overall and towards the end of the observed time span. All values were tabulated using Microsoft Excel (Microsoft Corporation, Redmond, Washington, USA).

For patients using rigid gas-permeable contact lenses, it was advised that they refrain from wearing their lenses for a minimum of 4 weeks before the tomographical examination. In the case of patients wearing soft contact lenses, they were requested to abstain from wearing them for at least 2 weeks prior to the examination.

To evaluate the postoperative development of CDVA, TP, Rmin, corneal astigmatism, and Kmax, paired t-test was performed, using the statistical software of IBM®-SPSS (International Business Machines Corporation, Armonk, New York, USA). The level of significance was set at 5%.

## Results

The gender distribution of the study population consisted of 41 male and 12 female patients, with an average age of 33 years (minimum = 19 years, maximum = 59 years). A total of 74 eyes were examined, with 35 being right eyes and 39 being left eyes. Among the patients, 21 individuals received treatment in both eyes.

The average time to final postoperative visit was 56 months (minimum = 28 months; maximum = 81 months). At an intermediate visit on average 12 months (minimum = 8 months, maximum = 17 months) prior to the most recent postoperative visit, the tomographical examination was performed in 24 eyes (9 right eyes, 15 left eyes) of 19 patients.

Preoperative CDVA was 0.23 logMAR (SD = 0.22), preoperative Rmin was 6.2 mm (SD = 0.7 mm), preoperative corneal astigmatism was 3.5D (SD = 2.0D), preoperative Kmax was 54.9D (SD = 6.4D) and preoperative TP was 463.1 µm (SD = 37.3 µm).

Postoperatively 53 (= 72%) eyes showed an improvement of visual acuity and 21 (= 28%) eyes showed a deterioration of visual acuity, whereas 16 (= 76%) eyes showed a deterioration of + 0.1 logMAR or less. In 3 (= 14%) eyes a deterioration of + 0.15 logMAR was observed, in 1 eye (= 5%) + 0.2 logMAR and in 1 eye (= 5%) + 0.3 logMAR.

**Fig. 1:**
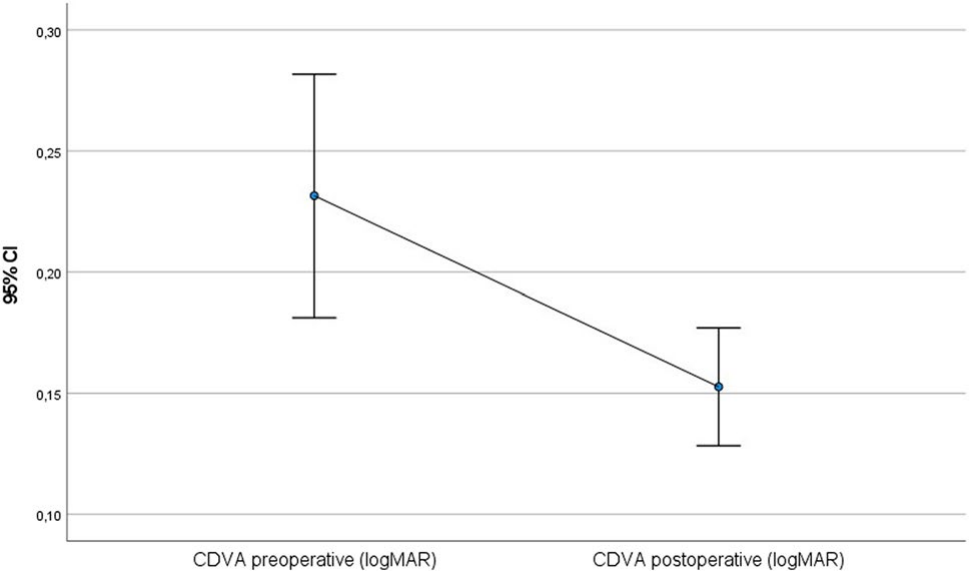
Comparison of pre- and postoperative corrected distance visual acuity (CDVA in logMAR) visualised via boxplot

**Fig. 2:**
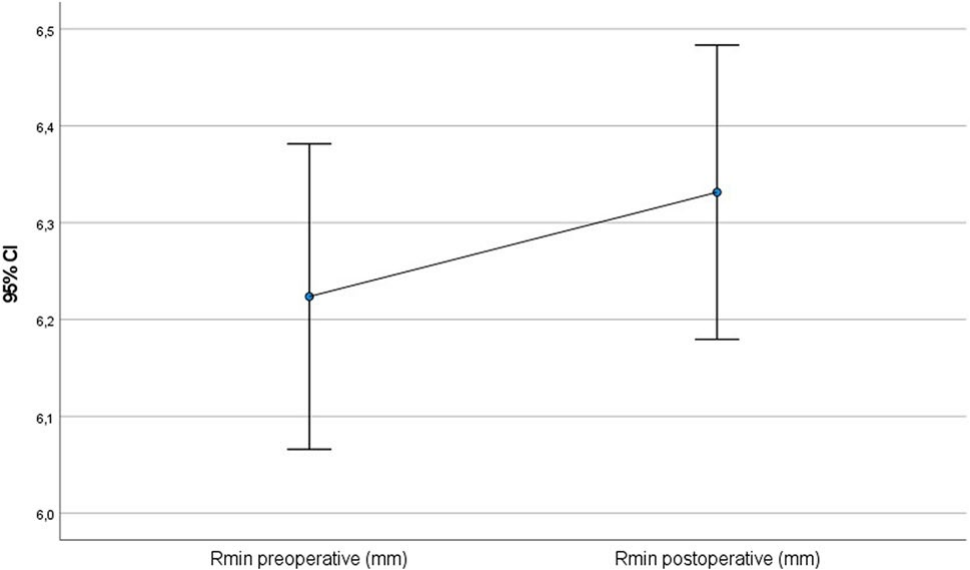
Comparison of pre- and postoperative minimum anterior radius (Rmin in mm) visualised via boxplot

**Fig. 3:**
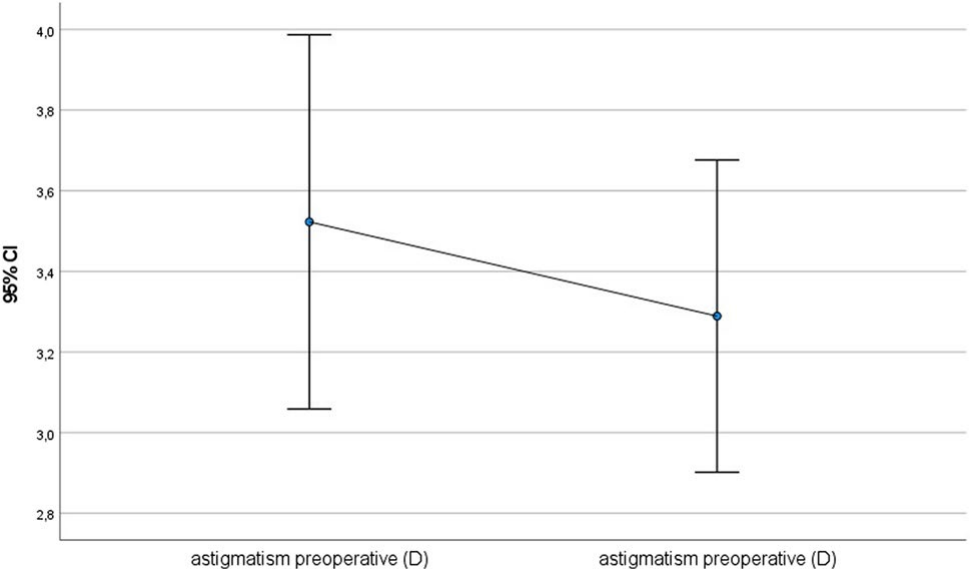
Comparison of pre- and postoperative astigmatism (D) visualised via boxplot

**Fig. 4:**
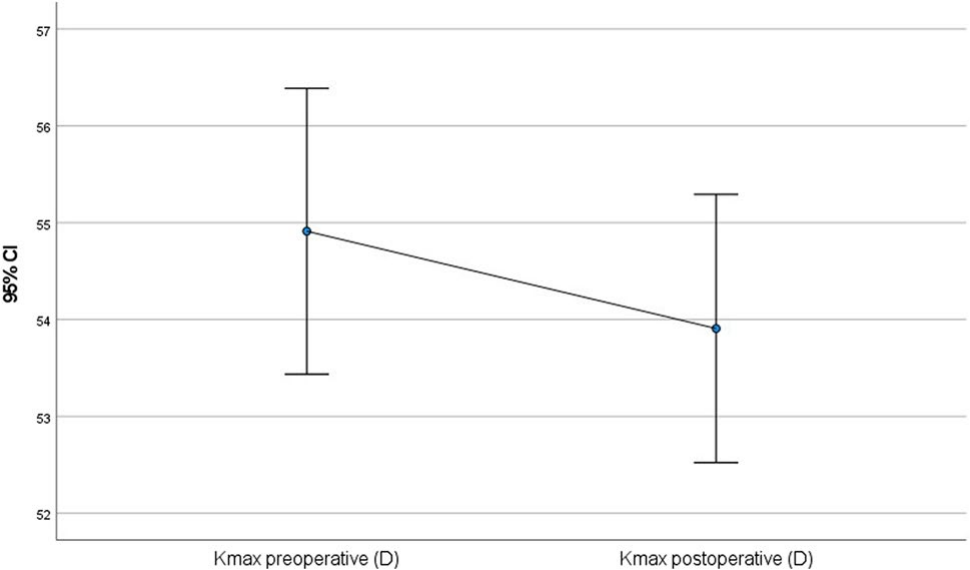
Comparison of pre- and postoperative maximum anterior keratometry (Kmax in D) visualised via boxplot

**Fig. 5:**
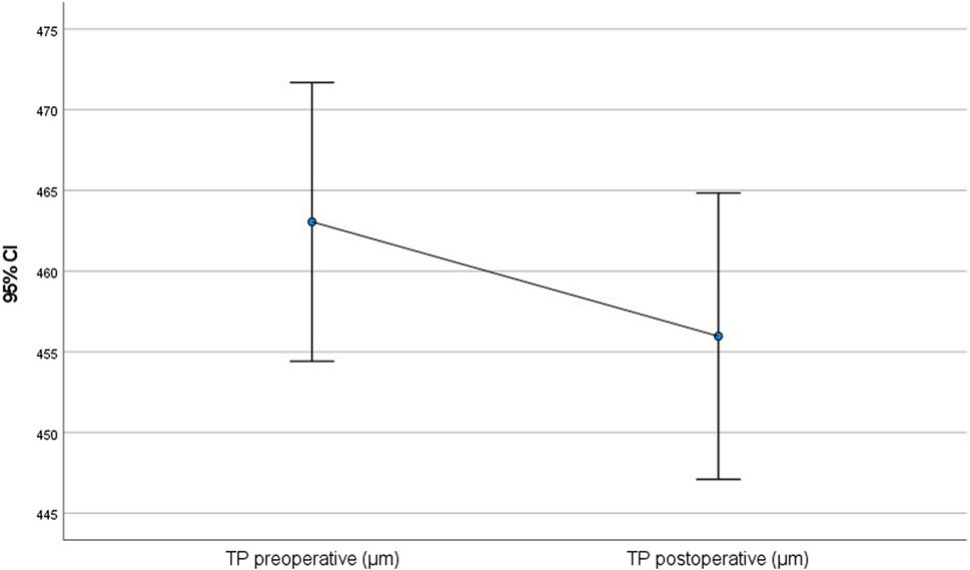
Comparison of pre- and postoperative thinnest corneal point (TP in µm)

All of the following values improved statistically significantly after 56 months (paired t-test): postoperative CDVA was 0.15 (SD = 0.10; *p* = 0.001) (Fig. 1), postoperative Rmin was 6.3 mm (SD = 0.7 mm; *p* < 0.001) (Fig. 2), postoperative corneal astigmatism was 3.3D (SD = 1.7D; *p* = 0.03) (Fig. 3) and postoperative Kmax was 53.9D (SD = 6.0D; *p* < 0.001) (Fig. 4). Postoperative TP decreased by 7.1 µm (SD = 38.3 µm; *p* < 0.001) (Fig. 5). All values are shown in Table 1.

**Table 1:** Pre- and postoperative tomographical data analysed via paired t-test

	Preoperative		Postoperative		Significance
CDVA (logMAR)	0.23	SD = 0.22	0.15	SD = 0.10	*P* = 0.001
Rmin	6.2 mm	SD = 0.7 mm	6.3 mm	SD = 0.7 mm	*p* < 0.001
Corneal Astigmatism	3.5D	SD = 2.0D	3.3D	SD = 1.7D	*P* = 0.03
Kmax	54.9D	SD = 6.4D	53.9D	SD = 6.0D	*p* < 0.001
TP	463.1 µm	SD = 37.3 µm	456.0 µm	SD = 38.3 µm	*p* < 0.001

Data including corrected distance visual acuity (CDVA in logMAR), minimum anterior radius (Rmin), Corneal Astigmatism, maximum anterior keratometry (Kmax) and thinnest corneal point (TP)

The subgroup analysis of 24 eyes from 19 patients, who underwent an examination 12 months prior to their postoperative visit, revealed no statistically significant changes in tomographical values compared to their final postoperative data. The paired t-test analysis showed p-values of 0.27 for TP (thinnest corneal point), 0.67 for Rmin (minimum radius), 0.72 for corneal astigmatism, and 0.72 for Kmax (maximum anterior keratometry).

Treatment failure was rigorously defined by a total increase of Kmax > 1.0D during the complete postoperative follow-up and occurred only in 3 eyes (= 4%). All three cases had initial Kmax < 55D. Progression of Kmax was still < 2D (1.5D, 1.1D, and 1.4D). The age of these three patients ranged between 23 and 30 years.

## Discussion

According to the Bunsen-Roscoe law, the photochemical effect on a tissue is determined by the product of the physical intensity of the flash and its duration. However, it should be noted that this law may not be entirely applicable to corneal tissue due to the limited number of crosslinking points within the cornea and the capacity for oxygen diffusion within the stroma [11, 12].

In ex vivo studies, it has been demonstrated that adequate corneal stiffness can be achieved even with irradiation levels as high as 45mW/cm2 for 2 min [13]. Shorter treatment durations offer increased patient comfort and allow for higher patient throughput. Importantly, the incidence of early negative effects, such as sterile infiltrates (7%), delayed epithelial healing (6%), bacterial infection (3%), and stromal scars (2%), does not appear to be higher when compared to conventional CXL following the Dresden protocol [10, 14].

Previous studies have proven similar efficacy of ACXL (18mW/cm^2^ for 5 min) compared to CXL following Dresden protocol after a follow-up time of six months to two years [15–19]. Further studies investigated very high-energy ACXL with irradiation of 30mW or more [20, 21]. Numerous long-term observations have been reported investigating ACXL with irradiation of 9mW/cm^2^ for 10 min [22–26]. By increasing the irradiation energy and thus lowering exposure time a more anterior demarcation line in OCT has been shown, raising the debate of less effective crosslinking, especially since a faster depletion of available oxygen molecules due to the increased irradiation is suspected [27, 28]. Recent research has been going into improving accelerated crosslinking efficacy by modifying the amount of available oxygen to compensate for these effects [29]. It is therefore of ongoing importance to prove the real-life efficacy of the different high-energy ACXL settings in stabilizing Keratoconus over prolonged observation times.

To the best of our knowledge, the present work is the first one to show the efficacy of HPMC-riboflavin ACXL with irradiation of 18mW/cm^2^ for 5 min after a long-term follow-up of 56 months. Hashemi et al. evaluated long-term follow-up after Dextran-riboflavin ACXL (18mW/cm^2^) showing a reduction of Kmax of 0.36D to 0.37D. Our results show a reduction of Kmax of 1D after 56 months. Hashemi et al. used Dextran-solution eye drops instead of HPMC-solution eye drops to apply riboflavin [30].

As mentioned earlier, accelerated corneal crosslinking (ACXL) has been associated with a shallower demarcation line compared to the conventional crosslinking protocol [31]. However, the use of hydroxypropylmethylcellulose (HPMC) solution in ACXL allows for faster intrastromal diffusion of riboflavin, resulting in a deeper demarcation line. This deeper demarcation line has been shown to lead to greater topographic flattening compared to ACXL with dextran solution [27, 32]. Studies have also reported greater topographic flattening after ACXL in patients with initially steeper corneas (Kmax 54.0D or more) [28, 33, 34]. Additionally, ACXL appears to be more effective in preventing progression in patients with advanced keratoconus [35]. However, concerns have been raised about the reduced total amount of generated crosslinks in ACXL, leading to suspicion of reduced long-term stability.

Our study is limited by the retrospective design with a lack of randomization and the absence of a control group due to the fact that our center switched over to ACXL early and completely, never providing conventional CXL and ACXL at the same time.

Our study findings indicate a statistically significant improvement in both functional and tomographical outcomes following high-energy ACXL (18mW/cm2 for 5 min) even after a long-term follow-up period of 56 months. Moreover, we observed stable conditions in patients with previously progressive keratoconus, including during the final year of the observation period. The treatment failure rate, defined as an increase in Kmax greater than 1D during the 5-year follow-up, was 4%. It is important to note that the limitations of our study, including the retrospective design and absence of a randomized control group, should be taken into consideration when interpreting these findings.

HPMC-riboflavin ACXL with irradiation of 18mW/cm^2^ for 5 min is an effective and safe treatment in patients with progressive keratoconus with no signs of fading stability for at least 4–5 years after surgery.
